# Non-repeatable science: assessing the frequency of voucher specimen deposition reveals that most arthropod research cannot be verified

**DOI:** 10.7717/peerj.1168

**Published:** 2015-08-06

**Authors:** Shaun Turney, Elyssa R. Cameron, Christopher A. Cloutier, Christopher M. Buddle

**Affiliations:** Department of Natural Resource Sciences, McGill University, Ste-Anne-de-Bellevue, QC, Canada

**Keywords:** Vouchers, Entomology, Philosophy of science, Verifiability, Biodiversity, Taxonomy, Museum collections

## Abstract

Scientific findings need to be verifiable and grounded in repeatability. With specimen-level research this is in part achieved with the deposition of voucher specimens. These are labeled, curated, data-based specimens that have been deposited in a collection or museum, available for verification of the work and to ensure researchers are calling the same taxa by the same names. Voucher specimens themselves are the subject of research, from the discovery of new species by taxonomists to ecologists documenting historical records of invasive species. Our objective was to quantify the frequency of voucher specimen deposition in biodiversity and community ecology research through a survey of the peer-reviewed literature about arthropods, from 1989 until 2014. Overall rates of voucher deposition were alarmingly low, at under 25%. This rate increased significantly over time, with 35% of papers reporting on vouchers in 2014. Relative to the global mean, entomological research had a significantly higher rate of voucher deposition (46%), whereas researchers studying crustaceans deposited vouchers less than 6% of the time, significantly less than the mean. Researchers working in museums had a significantly higher frequency of voucher deposition. Our results suggest a significant culture shift about the process of vouchering specimens is required. There must be more education and mentoring about voucher specimens within laboratories and across different fields of study. Principal investigators and granting agencies need a proactive approach to ensuring specimen-level data are properly, long-term curated. Editorial boards and journals can also adopt policies to ensure papers are published only if explicit statements about the deposition of voucher specimens is provided. Although the gap is significant, achieving a higher rate of voucher specimen deposition is a worthy goal to ensure all research efforts are preserved for future generations.

## Introduction

Deposition of voucher specimens embeds one’s research in the past and ensures its place in the future. Vouchers are representative specimens of a species that are physically deposited in a collection or a curated museum or institution and are accompanied by metadata and (optionally) DNA samples. It is imperative that other researchers have access to these vouchers as they are the fundamental unit of study for science that is grounded in specimen-based research (Huber, 1998; Suarez & Tsutsui, 2004; Astrin, Zhou & Misof, 2013). Vouchers come in several kinds, including those that are used to describe newly discovered species, known as “type” or “name-bearing” specimens. These vouchers, and their deposition, are governed worldwide by the *International Code of Zoological Nomenclature* (Dubois, 2010). The use and storage of “type” specimens, the development of taxonomic keys and identification tools, mitigation of past nomenclature errors and verification of future specimens would not be possible without vouchers. Verification vouchers in arthropod research are those that are deposited in a curated collection and are used as the basis of identification and verifiability of that particular research. Vouchers are primarily used to verify specimen identity, so that researchers can validate the work of others, or confirm their own specimen identifications (Schilthuizen et al., 2015).

As was argued decades ago by Knutson (1984), voucher specimens are central to repeatability in science. Whether taxonomy or systematics, evolution or ecology, comparisons among published studies require researchers to use the same name to refer to the same organism through time and vouchers are the basis of these comparisons (Lane, 1996; Danks, 1991; Suarez & Tsutsui, 2004). Martin (1990) discussed how voucher specimens ensure new species concepts can be applied to past research, thus further highlighting the need for proper deposition of vouchers. The advent of molecular tools and advances in data analysis and databases has led to species reclassification and identification at an ever-increasing rate, furthering the need for vouchers (Pleijel et al., 2008; Astrin, Zhou & Misof, 2013).

“Good science” must be both repeatable and verifiable. Vouchers allow for the verifiability of field studies involving the collection of specimens. Methods can be repeated and findings can be confirmed or challenged if desired, with vouchers providing “proof” of the investigator’s scientific claims, lending the claims greater weight. Vouchers are essentially representative data permanently stored in a museum or collection, and as such, new researchers can investigate scientific questions not addressed by the original collectors. Biodiversity and conservation studies represent a potentially large investment of money, time and effort. If proper vouchers are not prepared, a large part of this investment may be lost (Wheeler, 2003; Funk et al., 2005). The ability to retrospectively increase the depth of an investigation allows the expansion of our knowledge of the ecology, history, biogeography, conservation, morphology and genetics of collected species, all without any further specimen collection (e.g., Gardner, Heinsohn & Joseph, 2009). Most importantly, the opportunity for large verifiable and replicable meta-analyses of collected specimens is only possible with the existence of vouchers. In taxonomy and systematics, researchers can use vouchers and their accompanying data to develop new species descriptions, new or improved taxonomic keys, and improve phylogenies of existing taxa (Dekoninck et al., 2011). Important discoveries about the effects of anthropogenic change (climate change, habitat modification, etc.) on ecological communities, as well as discoveries about invasive species, species extinctions or global biodiversity, would all be unverifiable without vouchers (Dubois, 2010).

The use of vouchers expands beyond the scientific community: vouchers are useful to society as a whole, despite being largely underappreciated in this context (Suarez & Tsutsui, 2004). The use of voucher collections can help track and determine the source of disease outbreaks and environmental contaminants to aid in public health (Graham et al., 2004; Suarez & Tsutsui, 2004). The agricultural sector can also benefit from vouchers by an improved understanding of pest species movements, history and ecology (Lane, 1996; Suarez & Tsutsui, 2004). Vouchers allow the monitoring of species involved in the ecological functions that provide ecosystem services for the benefits of society. A detailed understanding of the historical changes of these services and the species involved will allow them to be better conserved (Lane, 1996; Suarez & Tsutsui, 2004). Therefore, in addition to being a crucial component of good repeatable science, vouchers can also serve as important historical and forensic evidence for societal benefit.

There is a longstanding tension between the important role of vouchers in science and the need to protect endangered species. An ethical dilemma arises: does the scientific worth of a voucher outweigh the life of an individual organism (Dubois & Nemésio, 2007; Dubois, 2009; Dubois, 2010)? In the case of arthropod research, voucher specimens are generally less contentious, probably because an individual arthropod is typically seen as a lesser organism compared to a vertebrate in the eyes of the public and researchers.

In addition to a species name (or higher taxonomic level), voucher specimens share the following qualities: they are deposited in a curated museum or institution, are accessible to other researchers and include meta-data (e.g., habitat, date, geo-reference), a unique identifier code, a museum or institution code, and the name of the collector(s) and the individual(s) who did the identification (Martin, 1990; Lane, 1996; Wheeler, 2003). Without appropriate meta-data accompanying vouchers there would be no way for other scientists to use the vouchers as, (1) they would be unable to access or find the physical specimen and (2) they would not know the geographical and ecological context of the specimen.

Other types of specimen records (e.g., DNA, photographs, sound recordings) are not vouchers strictly speaking, although they can play a similar or complementary role, especially when made available online. Although these types of non-voucher records are useful, voucher specimens play a unique and critical role in biological science. The possibilities for the extraction of information from a physical specimen are far greater than the information available from a single photograph or DNA sequence (Dubois & Nemésio, 2007). Valkiūnas et al. (2008) argued that whenever DNA samples are deposited, a physical specimen should be deposited along with it. This will ensure, especially if the physical specimen is verified by an expert taxonomist, that DNA sequences which are deposited in online databases such as GenBank are based on accurate identifications of specimens.

In order to integrate the results of all relevant studies into the broader environmental and ecological context, data must be made readily available to both the public and scientific communities (Schilthuizen et al., 2015). As such, there is a move to increase public access to online data from scientists, and accordingly more researchers can publish voucher-related information online to sites such as FigShare, GenBank^®^, PANGAEA^®^ and others (Pleijel et al., 2008). Although as previously mentioned this does not negate the need for the physical specimen as well. As these various databases develop, a more expanded and detailed network linking all voucher-related information is necessary in order to produce higher-quality science, more confident results and improved accuracy and availability of information (Danks, 1991; Rowley et al., 2007; Pleijel et al., 2008; Astrin, Zhou & Misof, 2013).

There is a long history of deposition of arthropod vouchers (e.g., the insect collection of Carl Linnaeus held by the Linnean Society of London.). This is perhaps because their body structure lends easily to pinning and preservation using relatively simple techniques compared to other classes of animals (Huber, 1998). This history of voucher deposition can lead to important discoveries such as revealing century-old patterns in insect species status, records and composition (Parmesan et al., 1999; Schlick-Steiner, Steiner & Stefan, 2003; Dekoninck et al., 2011), plant–pollinator interactions (Scheper et al., 2014) and even the paleontological past (Graham et al., 2004). Curated arthropods are the currency of arthropod systematics, and museum specimens form the basis of significant taxonomic revisions and new species discoveries (Dekoninck et al., 2011). Additionally, arthropods are understudied, hyper-diverse, and the differences between species can be subtle. Vouchers specimens help disentangle this complexity (Rowley et al., 2007) especially when dealing with cases of different casts or morphs amongst a single species, sexual dimorphism or dichromatism and ontogenetic variation within a species lifecycle (Dubois, 2010). Though many taxonomic keys exist, there is always a need for better, more accessible, and up to date resources for non-specialists (e.g., *Canadian Journal of Arthropod Identification*). Producing such publications is extremely difficult, if not impossible, without voucher specimens.

Given the importance of vouchers, we quantified the frequency at which researchers deposit voucher specimens, with a focus on arthropod research. To our knowledge, only one paper has investigated this: Bortolus (2008) found that 50 out of 80 surveyed papers in ecology journals did not have any “*supporting information justifying or guaranteeing the correct identification of the organisms studied or manipulated*” (Bortolus, 2008: p. 114). This supporting information does not refer solely to voucher specimens, and we assume even fewer papers involved specimens that were deposited in a curated museum or collection. In contrast, we are anecdotally aware that many entomology journals, and entomology colleagues in systematics and taxonomy, regularly deposit and/or refer to voucher specimens. Briefs have been prepared that outline the methods and importance of vouchers in entomology research (Huber, 1998; Wheeler, 2003). Our goal was therefore to determine whether arthropod researchers working in biodiversity and community ecology related fields are depositing vouchers. To do so, we carried out a detailed survey of published literature to determine the factors that relate to frequency of voucher specimen deposition. We then tested the frequency of voucher deposition over time, by taxon, discipline, institution type, and by the number of taxa studied in the research papers.

## Materials & Methods

### Paper selection

The papers included in our meta-analysis were selected from Scopus, a database of peer-reviewed literature. We searched for papers in which the title, abstract or keyword contained “arthropod*,” “entomology*,” “arachnid*,” “spider*,” “insect*,” “crustac*” and “myriapod*.” The title, abstract or keyword also needed to contain “biodivers*,” “communit*,” “species richness” or “assembl*.” The search was open to all Scopus subject areas. These search terms were selected with the objective of finding papers in which the authors collected and identified arthropod specimens belonging to multiple taxa, with an aim to exclude most of the purely genetic and taxonomic single species papers and remain within the scope of biodiversity and community ecology. For feasibility we narrowed our paper selection to those published every five years beginning in 1989 (1989, 1994, 1999, 2004, 2009 and 2014).

**Figure 1:**
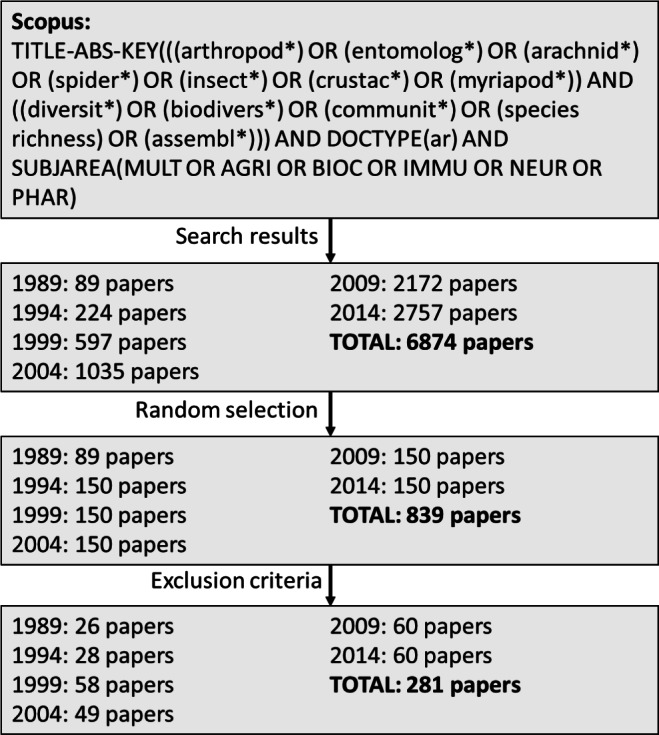
Selection criteria cascade used to generate the papers included in this analysis. The original search in Scopus using our search terms generated a total of 6,874 papers across the six years. These were then subjected to random selection and finally exclusion criteria which generated a total of 281 papers.

The number of papers returned by the search terms was highly variable and increased over time, ranging from 89 papers in 1989 to 2757 in 2014 (Fig. 1). From each year, 150 papers were randomly selected using a random number generator, with the exception of 1989, for which all papers were included. Each of these papers was then subjected to two selection criteria: (1) the study involved the collection of arthropod specimens from the field, and (2) three or more taxa of arthropods were collected and identified. Both of these criteria aimed to focus our results to include those papers dealing exclusively with original work which would require specimen identifications and biodiversity or community ecology research. It was assumed that purely taxonomic research or studies dealing with one or two species would either be already using vouchers for their work/data, or would already have the culture for voucher deposition ingrained within their discipline. Journals in taxonomic fields are also more likely to possess guidelines which require voucher deposition, particularly those dealing with systematics (Wheeler, 2003). Of the randomly selected subset, 33.5% of papers passed the inclusion criteria and were included in the final analysis (Fig. 1).

### Data collection

For each of the papers included in the final analysis, we collected data concerning the study and the journal in which it was published. For the study, we recorded: the number of authors; the institution type of the lead author (i.e., university, museum, private, non-profit, government or other); the biome type (i.e., terrestrial, marine, freshwater); the taxonomic resolution(s) to which the specimens were identified (e.g., family, genus, species); the number of taxa identified; the number of specimens collected; the class-level taxa collected (Arachnida, Insecta, Crustacea, Myriapoda and other); the insect order collected (Coleoptera, Diptera, Lepidoptera, Hymenoptera and other); and whether or not non-arthropod specimens were collected. We recorded whether or not the paper included a specific mention of their deposition of voucher specimens and, if so, the type of information attached to vouchers (i.e., digital, DNA, whole/part of specimen, other). We recognize that only whole/part of specimens represent “true” vouchers; however, since other types of specimen records can complement and serve a purpose similar to a voucher, we decided to include them in our analysis.

In order to extract data concerning the journals in which the papers were published, we used the R package CITAN (R Core Team, 2014; Gagolewski, 2011). We recorded the Scopus All Science Journal Classification codes (ASJC) for each journal—this is a code designating the scientific discipline to which each journal contributes. Molecular biology, cell biology, biochemistry and microbiology were grouped together as Cell Biology. In our analysis, we included this grouped ASJC, along with Entomology (ASJC: “Insect Science”), EcoEvo (ASJC: “Ecology, Evolution, Behavior and Systematics”), and Genetics (ASJC: “Genetics”). We also extracted from each journal the 2011 Scopus Source Normalized Impact per Paper (SNIP; Leydesdorff & Opthof, 2010), if available, and whether or not the journal is categorized by Scopus as Open Access.

Of the journals included in our analysis, we randomly selected a subset from each of the four categories; Entomology (10 journals), EcoEvo (10), Cell Biology (4), and Genetics (5). The Author Guidelines were examined for each of the journals included under each category and we recorded whether or not vouchers were required, recommended, or not mentioned as a requirement of publication.

### Data analyses

The data were analyzed with general linear models (GLM), using the *glm* function in R. The response variable, voucher inclusion, was a binomial variable (yes/no) and so a binomial family was used. In the full model, the following explanatory variables were included: publication year, number of authors, author institution type (university, government, non-profit, private, museum), number of species, number of specimens, biome (terrestrial, marine, freshwater), Class (Crustacea, Insecta, Arachnida), insect order (Hymenoptera, Diptera, Lepidoptera, Coleoptera) whether non-arthropods were collected, SNIP, whether the journal is Open Access, and the ASJC category (Entomology, Cell Biology, EcoEvo, Genetics).

For each categorical variable (author institution type, biome, class, insect order, ASJC category), each category was included as a separate explanatory variable, since within each categorical variable papers could belong to more than one category. Each category was thus compared by the model to the mean voucher proportion rather than to the voucher proportion of other categories of the same type. Some categories were excluded from the model because few papers fell under these categories (Table 2).

The full GLM was subjected to backward selection using the *stepAIC* function in R. The final model was repeated as a mixed effects GLM using the R package *lme4* (Bates et al., 2014). The journal was included as a random effect and all other variables from the final GLM were included as fixed effects. This was in order to account for any violation of the GLM assumption of independence of observation due to some papers having been published in the same journal.

## Results and discussion

Of the 281 papers surveyed, less than 25% (66 papers) stated that they deposited voucher specimens or other specimen records (Table 1). Of these studies, 56 of these deposited “true” vouchers (whole/part specimen) and the remaining 10 studies deposited only DNA and/or photographs. This is a worrying statistic, given that vouchers are the cornerstone of repeatability in specimen-based arthropod research. For more than 3/4 of the papers we read, with only the peer-reviewed paper in hand, nobody would be able to verify the findings, at least, not without a lot of legwork. Future researchers must therefore assume the authors were correct in their identifications, which could pose serious consequences should there be errors or changes in taxonomy.

**Table 1:** Percentages of papers which included vouchers or other specimen records based on the different categories and category types. “Total” refers to the accepted papers which fit a given category. “Vouchers” refers to the number of accepted papers which fit a given category and included vouchers in their study. “Percent” is the percentage of accepted papers in the given category which included vouchers. The “Total accepted” category type refers to all 281 papers which were included in the study (Fig. 1).

Category type	Category	Total	Vouchers	Percent (%)
**Habitat type**	Marine	61	4	6.6
	Freshwater	63	9	14.3
	Terrestrial	163	53	32.5
**Class**	Myriapoda	9	2	22.2
	Insecta	176	47	26.7
	Crustacea	102	6	5.9
	Arcachnida	76	20	26.3
**Insect taxa**	Coleoptera	94	20	21.3
	Hymenoptera	63	19	30.2
	Diptera	82	14	17.1
	Lepidoptera	41	11	26.3
	Other	82	16	19.5
**Species level**	Species	247	61	24.7
	Above species	34	5	14.7
**Institution type**	University	194	49	25.3
	Museum	17	8	47.1
	Government	58	9	15.5
	Private	15	5	33.3
	Non-profit	7	3	42.9
	Other	2	0	0.0
**Year**	1989	26	3	11.5
	1994	28	1	3.6
	1999	58	11	19.0
	2004	49	11	22.4
	2009	60	19	31.7
	2014	60	21	35.0
**Number of taxa**	0–5	19	1	5.3
	6–15	27	4	14.8
	16–30	33	10	30.3
	31–60	71	19	26.8
	>60	126	31	24.6
**Journal type**	Genetics	10	3	30.0
	Entomology	70	32	45.7
	EcoEvo	107	25	23.4
	Cell biology	11	3	27.3
**Total**		281	66	23.5

**Figure 2:**
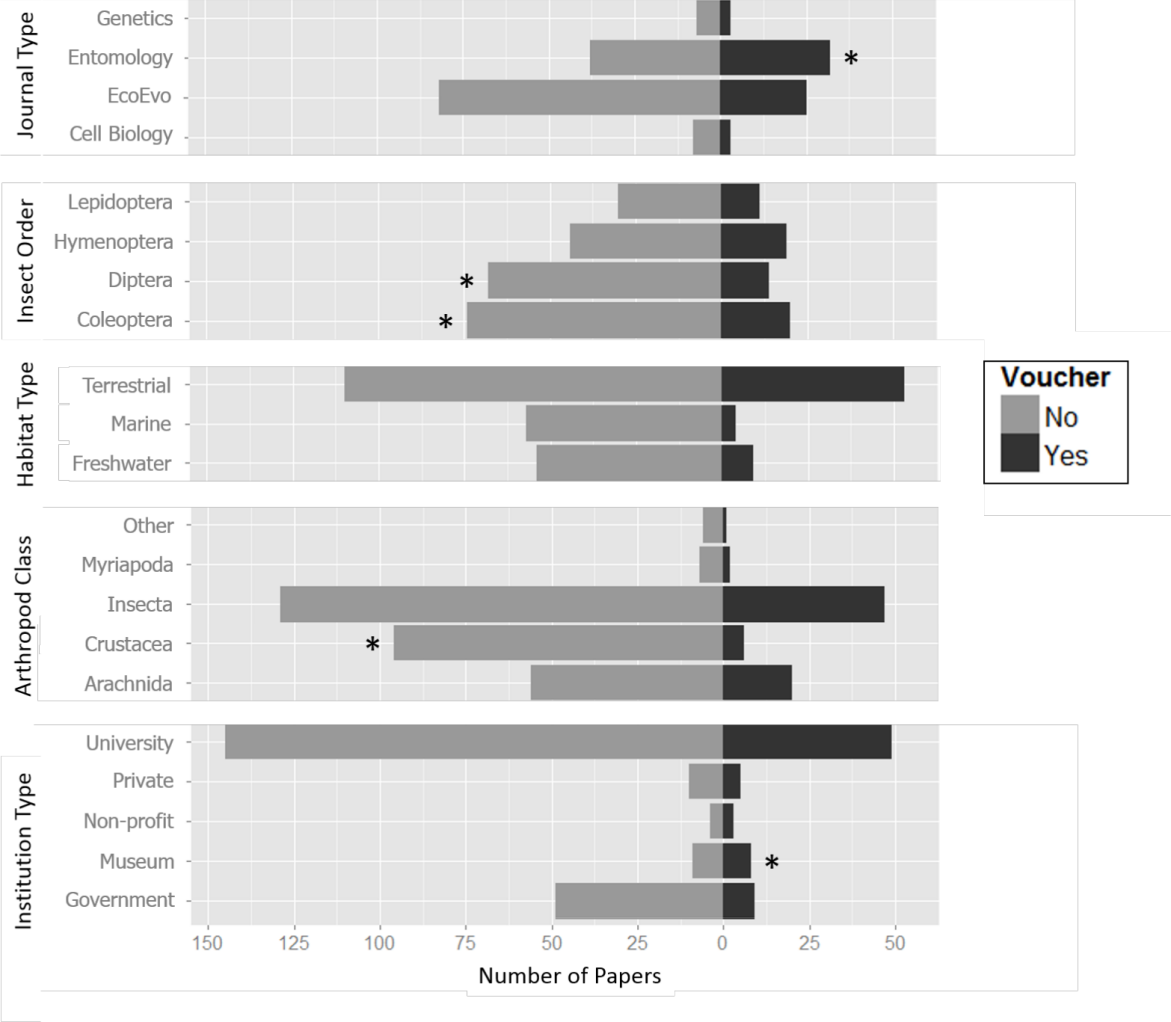
The proportion of studies which did (Yes) and did not (No) produce vouchers in categories among a total of 281 papers randomly selected from a Scopus database and subjected to selection criteria. Categories shown here are for journal type, insect order, habitat type, arthropod class and institution type. Significance values as compared to the global mean (indicated with an *) include Entomology (*P* = 0.018) and Museums (*P* = 0.049) which do have a high tendency to produce vouchers, and Diptera (*P* = 0.056), Coleoptera (*P* = 0.043), and Crustacea (*P* = 0.0004) which do not.

The frequency of voucher specimen deposition varied by discipline (as defined by Scopus journal classification). Entomology journals had a significantly higher frequency of voucher deposition compared to the global mean (Fig. 2; Tables 1 and 2), which resonated with our anecdotal experiences working with entomologists. In fact, Wheeler (2003) examined the policies of various journals, including both Canadian and international entomological journals, and their stance on voucher deposition. He noted that of all the journals considered, only those with a strong focus in systematics require vouchers to be deposited whereas all others (with the exception of a few) do not mention vouchers in their policies or only mention them as a recommendation (Wheeler, 2003). Despite this, even within entomology journals, the rate of voucher specimen deposition was still below 50% (Table 1). The other disciplines (EcoEvo, Genetics, Cell Biology) fared poorly compared to Entomology (Fig. 2). Additionally, the SNIP (Impact Factor) and whether or not the journal was Open Access had no significant effect on the proportion of voucher deposition.

**Table 2:** A binomial family generalized linear model of arthropod vouchers (presence/absence) after backward stepwise model selection. A total of 281 papers were included in the model, randomly selected from a Scopus search and subjected to inclusion criteria (Fig. 1). The analysis was repeated as a mixed effects generalized linear model in which the journal for which each paper was published was included in the model as a random effect. Significant effects for either the generalized linear model (GLM) or the generalized linear mixed model (Mixed) are bolded.

**Coefficient**	Estimate		Standard error		*Z* value		*P* value	
	GLM	Mixed	GLM	Mixed	GLM	Mixed	GLM	Mixed
**(Intercept)**	−120.1	−119.6	5.025	1.026	−2.390	−11.652	0.0168	<0.0001
**Year**	**0.059**	**0.059**	**0.025**	**0.005**	**2.359**	**11.542**	**0.0184**	**<0.0001**
**Private**	1.111	1.170	0.697	0.791	1.595	1.479	0.1107	0.1391
**Museum**	**1.210**	**1.462**	**0.596**	**0.744**	**2.032**	**1.965**	**0.0422**	**0.0494**
**Number of taxa**	**0.013**	**0.001**	**0.006**	**0.0006**	**2.267**	**1.919**	**0.0234**	0.0550
**Crustacea**	**−2.033**	**−2.301**	**0.526**	**0.652**	**−3.867**	**−3.531**	**0.0001**	**0.0004**
**Entomology**	**1.046**	**1.232**	**0.376**	**0.525**	**2.782**	**2.347**	**0.0054**	**0.0189**
**Systematics**	0.531	0.658	0.360	0.472	1.475	1.392	0.1403	0.1638
**Open access**	0.915	1.067	0.566	0.725	1.616	1.471	0.1061	0.1413
**Diptera**	**−0.826**	**−0.944**	**0.405**	**0.494**	**−2.042**	**−1.910**	**0.0412**	0.0561
**Coleoptera**	**−0.833**	**−0.952**	**0.378**	**0.471**	**−2.202**	**−2.022**	**0.0276**	**0.0431**

Our own analysis of journal author guidelines agreed with the finding of Wheeler (2003). Of the 10 randomly generated journals under the discipline of “Entomology,” only three made mention of voucher deposition. Of these, none required vouchers to be deposited, but they only recommended it, except in the case of type-specimens. Those under the discipline of “EcoEvo” again made no mention of vouchers being required and only mentioned that DNA sequences, geographic coordinates and phylogenetic trees should be deposited in online databases. In some cases this is obligatory. In both the “Cell Biology” and “Genetics” disciplines, no reference was given to the requirements of vouchers except for one genetics based paper dealing with systematics.

Comparisons between the studied taxa supported this general pattern of entomology research having a stronger culture of voucher deposition as compared to other disciplines. The Class Insecta had the highest percentage of papers that included voucher specimens at 26.7%, followed closely by Arachnida (Fig. 2; Table 1). However, the proportions of voucher deposition among Insecta and Arachnida research were not significantly different from the global mean (Table 2). Within the “big four” insect Orders, Diptera (flies) and Coleoptera (beetles) had a significantly lower proportion of voucher deposition than the global mean (Fig. 2; Table 2). Interestingly, these were also the most studied Orders from our survey of the published literature (Table 1). The low rate of voucher deposition of these orders may be because some of their taxa are taxonomically challenging. Although vouchers are all the more important in cases when misidentification is likely, researchers are human and may avoid time-consuming or frustrating tasks. For example, many Diptera species are quite small, with some species measuring less than 1 mm in body length. Their tiny size renders them challenging to identify and pin, and often additional preparations are required to make flies suitable for voucher deposition (e.g., critical point drying).

Scientists studying crustaceans seldom deposited vouchers, with fewer than 6% of papers involving crustaceans reporting voucher production (Fig. 2; Table 1). The severe lack of voucher deposition observed may be partly explained by the method of preservation typically used within crustacean research, which involves the use of 70–90% ethanol (Huber, 1998), a bulkier method which requires more storage space. Similarly to Crustacea, arachnid specimens are also commonly stored using ethanol. Despite this, research involving arachnids showed a much higher proportion of voucher deposition. Crustacea is a very diverse group with many cryptic species (Radulovici, Archambault & Dufresne, 2010), which may discourage researchers from making vouchers because identification is challenging. However, the importance of voucher specimens becomes greater as the probability of incorrect or taxonomic revisions increases.

Conversely, terrestrial arthropod research was much more likely to involve voucher deposition than aquatic (marine or freshwater) arthropod research (Fig. 2; Table 1). Crustacea research is usually aquatic, so the lack of voucher deposition among aquatic studies may be explained by the relative lack of a voucher deposition culture among Crustacea researchers. It may be that the frequency with which vouchers are created depends more on the taxa involved and the norms of its corresponding discipline rather than the method of preservation or habitat type.

The rate of voucher deposition varied among the institute type of the lead author. Museum research showed a 47% voucher deposition rate, which was a significantly higher rate than the global mean (Table 2). This was followed closely by non-profits at 43% and private institutions at 33%, although neither of these voucher deposition rates were significantly greater than the global mean (Table 1). The number of papers produced by each of the aforementioned institute types was low (<20), which suggests that a higher sample size could have detected significant differences. The most abundantly encountered institutions in our meta-analysis were universities, with 194 total papers found, and only 25% of them generated vouchers (Fig. 2; Table 1). Institution type was evaluated solely by the primary author and this created a degree of bias by excluding the different institutions of contributing authors. Although this is potentially a source of error, we assume that the primary author would have the greatest input into the handling of specimens. It was not feasible to analyze all possible combinations of institution-types for multi-author papers.

The low rate of voucher deposition among papers produced by universities may be a function of budgetary constraints (cost of labeling, mounting, curating, mailing of specimens, etc.), limitations on space (e.g., housing of a multitude of specimens; Wheeler, 2003) and non-permanency of research topics in universities. Conversely, museums do not face these challenges to the same extent, though in recent years they too have been subjected to funding cuts (Danks, 1991; Lane, 1996; Suarez & Tsutsui, 2004). Museums are typically better equipped for the purposes of specimen storage and preservation and usually have a designated curator to manage the collection. Museums maintain reference collections and make them available to other researchers for identification and verification of specimens; one of the essential roles of vouchers (Suarez & Tsutsui, 2004). Museums play a crucial role in the conservation of vouchers and they must continue to receive recognition and monetary support for the costly functions they carry out. Museums are, and must be recognized as, large scientific infrastructures useful to both basic and applied sciences (Dubois, 2010).

**Figure 3:**
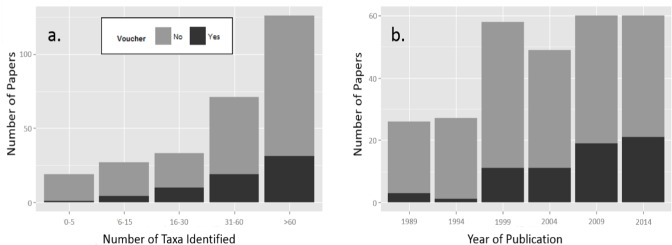
The proportion of studies which did (Yes) and did not (No) produce vouchers among a total of 281 papers randomly selected from a Scopus database and subjected to selection criteria. The number of vouchers increased significantly as compared to the global mean with (A) the number of taxa identified within the study (*P* = 0.055; Table 2) and (B) the year of study publication (*P* < 0.0001; Table 2)

As the number of arthropod taxa collected and identified within a study increased, the rate of reported voucher deposition also increased (Fig. 3A). Percentage of voucher deposition reached a peak at 30% for studies that identified between 16 and 30 taxa (Table 1). Following this peak, there was a slight decrease in the percentage of vouchers produced for studies that identified 30 taxa. The decrease in voucher deposition rate among high-diversity studies may relate to the tradeoff between cost, which increases with species number, and the need for verification, as misidentifications are more likely to occur when dealing with a greater diversity of taxa.

The encouraging news is that scientists are improving their frequency of voucher deposition over time, with a steady increase from 3.5% of papers in 1994 to 35% in 2014 (Fig. 3B). This effect proved strongly significant in our model. Despite this improvement over the years, 35% is still not high enough. Voucher deposition is necessary for the scientific soundness of any arthropod study that involves the collection and identification of specimens. There are rare cases in which vouchers may not be feasible or ethically-justifiable, but these cases require strong justification. Beginning in the 2000s, and increasing over time, researchers began depositing DNA sequences with their vouchers and sharing these on online databases. As genetic tools and online databases continue to become more accessible and user-friendly, we hope that the trend towards a greater voucher-deposition culture among arthropod researchers will continue.

## Recommendations and conclusions

Based on our results we have the following recommendations:

1. Responsibility for voucher creation should fall to all researchers involved, but especially to principal investigators. Principal investigators must take a proactive approach to voucher deposition and see this as a core activity of the research process rather than as an afterthought. It needs to be planned and budgeted for, and be central to all specimen-based research activities. It must be ensured that vouchers are stored in good condition within permanent, curated and accessible collections.
2. Supervisors need to mentor graduate students and other trainees about the importance of vouchers, and include a clear process for voucher deposition in their laboratories. Getting vouchers for all studies is the desired goal, but this will only be possible if there becomes more of a culture around vouchers across all laboratories and this starts with training and education.
3. Variation in vouchering by taxa needs to be reduced: having only 25% of papers report about voucher specimens is itself a low proportion, but having below 10% for some taxa is more alarming. We see no valid reason why entomologists should be better at submitting details of vouchers than those working on crustaceans: all arthropods are important arthropods. Perhaps clear policies or guidelines for other taxa are required, and we hope our work prompts such initiatives.
4. The difference between institution types highlights the need for closer collaboration among university researchers and museums. Instead of all universities setting up their own long-term collections, working with museums and their curators is ideal. We recognize that this will require additional funding of museum resources.
5. Curators and researchers need to continually push for sustainable funding for collections, and lobby administrators and granting agencies for support. Granting agencies must also create clear policies around vouchers, and see their importance at the same level as publications and curation of data. Field surveys are often costly, which means that each collected specimen has high financial value. By requiring that funded researchers deposit voucher specimens in permanent collections, funding agencies protect their financial investment. Ethics boards also have reason to require voucher deposition due to an element of respect for the collected animals. Researchers have a responsibility, especially for threatened or endangered species, to make maximal use of organisms that they have killed and collected in the name of science.
6. Editorial boards must adjust their own policies and guidelines around peer review and demand all papers related to specimen-level research include details about voucher deposition. Guidelines requiring vouchers will force authors to think about long-term storage of collected organisms and will change the culture surrounding voucher-deposition. Fundamentally, no study involving the collection of arthropods should be accepted by editors without either deposited vouchers or a strong justification for not depositing vouchers.

Now more than ever it is critical to increase the prevalence of vouchers in response to large scale climate and habitat modification which will invariably influence biodiversity worldwide. Our survey has highlighted an alarming gap and despite the increasing prevalence of voucher specimen deposition, we are very far from 100%. Establishing voucher specimens takes time and is not easy, but it is a worthy pursuit as vouchering is at the core of strong, repeatable science. Voucher collections will continue to strengthen our present-day arthropod science, connect it to research of the past, and provide important data for future research.

## Supplemental Information

10.7717/peerj.1168/supp-1 Data S1 Raw data
